# A moderate fat, low-energy dry expanded diet reduces gain in body condition score when fed as part of a post neutering weight-control regimen in growing pet cats*

**DOI:** 10.1017/jns.2014.48

**Published:** 2014-09-30

**Authors:** Nathaniel Spofford, Isabelle Mougeot, Denise A. Elliott, Ashlee Addleman, Sandra L. Lefebvre, Mansen Wang, Mingyin Yang, Alexandre Feugier, Vincent Biourge, Elizabeth M. Lund

**Affiliations:** 1 Banfield Pet Hospital, Portland, OR, USA; 2 Royal Canin Research Center, Aimargues, France; 3 WALTHAM^®^Centre for Pet Nutrition, Freeby Lane, Waltham on the Worlds, Melton Mowbray, Leicestershire, UK

**Keywords:** Overweight, Feline nutrition, Neutering, BCS, body condition score

## Abstract

Neutering of cats has been associated with significant weight gain in the weeks following surgery. The present study aimed to evaluate the effectiveness of a moderate fat, low-energy dry expanded diet in reducing weight gain in growing pet cats when fed as part of a weight-control regimen over the 6 months post-neutering. Cats in participating primary care veterinary hospitals were enrolled at neutering and assigned to receive one of the two dietary treatments based on the hospital of origin. Owners of cats in the treatment group were instructed to feed the trial diet at maintenance (324·7 kJ/kg BW^0·711^ per d). Instructions for the control group were to feed the cat's regular diet according to the manufacturer's recommendations. Body weight and condition were evaluated by veterinarians at enrolment, 2-weeks, and 1–4 and 6 months after surgery. Body condition score (five-point scale) was compared between enrolment and each subsequent visit, controlling for enrolment age and sex. Percentage change in body weight was evaluated via multivariate mixed modelling to account for repeated measures. A total of 187 cats (eighty-seven females and 100 males) with a mean age of 5·2 (sd 0·8) months and mean weight of 2·8 (sd 0·6) kg from fifty-one hospitals completed the trial. The odds of being scored as overweight were 4·1 times as great for cats in the control *v.* treatment groups (95 % CI 2·1, 8·2). Percentage change in body weight differed significantly with enrolment age (*P* = 0·007) and approached significance between diet groups (*P* = 0·08). Cats fed the trial diet had a significantly reduced incidence of overweight in the 6 months following neutering.

The reported combined prevalence of overweight and obesity in cats in the USA ranges between 16 and 35 %^(^^1^^–^^3^^)^. There is general agreement among investigators that the incidence of excess body weight in cats is increasing^(^^4^^)^. Indeed, the prevalence of overweight and obesity among cats evaluated at a large national network of US primary care veterinary hospitals increased by 90 % from 2007 to 2011^(^^3^^)^. Obesity has considerable impacts on the health of cats and is linked to a variety of health issues including diabetes mellitus, orthopaedic disease and non-allergic skin conditions^(^^5^^)^.

Castration and ovariohysterectomy are associated with increased risk of excess body weight^(^^2^^,^^6^^,^^7^^)^. Indeed, the post-neutering period is a time during which cats tend to gain weight if their food is not closely regulated^(^^8^^)^.This period presents an ideal opportunity to help prevent overweight and obesity through dietary intervention. Previous research suggests that feeding a low-energy-dense diet in carefully controlled meals is an effective means of reducing weight gain in neutered cats living in an experimental colony^(^^7^^)^. However, there is evidence that weight loss strategies that are effective under tightly controlled experimental conditions are less effective when implemented under conditions that better approximate real life^(^^9^^)^. The purpose of the present study was to follow client-owned and client-fed cats living at home in the 6 months following neutering to determine whether cats fed a moderate fat, low-energy dry expanded diet as part of a post-neutering weight-control regimen would be more likely to maintain their optimal body condition than cats fed their typical diet under the same conditions.

## Experimental methods

### Animals

Cats presented for castration and ovariohysterectomy at fifty-one veterinary hospitals in eighteen states across the contiguous USA were eligible to participate. These hospitals are a subset from a network of over 800 general practice hospitals. In order to facilitate enrolment hospitals were invited to participate based on the number of castration and ovariohysterectomy surgeries that were performed in 2010. Cats were eligible for enrolment if they were healthy, had an ideal body condition score (BCS) (3/5), were 4–7 months of age, were enrolled in a wellness care plan, were fed an exclusively dry food diet and could be fed separately from other household pets. Additionally, the cat's owner had to be willing and able to commit to long-term follow up, including regular completion of weekly online surveys and six in-hospital follow-up evaluations. Eligibility was determined by the veterinarian as well as through review of medical records and responses to an online enrolment survey completed by the cat's owner. The total of 253 cats were recruited to enable detection of a minimum difference of 10 % increase in body weight between diets fed, a conservative treatment difference based on previous research^(^^7^^,^^10^^)^. The study protocol was approved by the Royal Canin Committee for animal ethics and welfare and complied with Mars' Animal Welfare Policy. Staff at participating hospitals were trained on the study protocol via printed training materials and individual telephone consultations. Written informed consent was obtained from the owners of all participating animals.

### Study protocol

Cats were assigned to study groups based on the hospital at which they received veterinary care. A random-number scheme was used to assign participating hospitals to treatment or control groups. Owners of cats enrolled at hospitals in the treatment group were instructed to exclusively feed a moderate fat, low-energy dry expanded diet formulated for neutered cats (moisture 16·5 g, protein 105·0 g, crude fat 30·0 g, crude fibre 27·9 g, ash 30·6 g, non-fermentable extract 90·0 g, metabolisable energy 13 075 kJ/kg) measured according to written feeding guidelines based on the cat's present body weight. Written and verbal instructions were provided for owners to transition their cat from the food fed prior to surgery to the trial diet over a period of 1 week after neutering. Owners of cats enrolled at hospitals in the control group were instructed to exclusively feed their cat's regular food, measured based on the cat's present body weight according to the manufacturer's recommendations. Both groups received written instructions emphasising the importance of feeding their cat in measured amounts.

Cats were enrolled in the study on the day of castration or ovariohysterectomy. Follow-up visits were conducted 2-weeks and 1–4 and 6 months after enrolment. Data collected at enrolment visit included age in months, sex, indoor/outdoor exposure, general activity level and pre-enrolment diet. Data collected at all visits (i.e. enrolment and follow-ups) included weight and BCS, which were entered into a proprietary practice management system for later access by the research team. Diet compliance, diet quantity, access to additional food sources and any medications administered during the study were monitored via a weekly online survey completed by the owner. Online monthly surveys were completed to assess palatability (food acceptance), apparent digestibility and satiety of the cat's present diet as well as the perceived impact on the cat's skin and hair.

In order to optimise protocol compliance and the likelihood of study completion, a reminder was emailed to participants 10 d prior to each visit due-date and follow-up phone calls were made by the investigators if a visit had not been completed by the target date. Participants were removed from the study if they missed more than one follow-up visit or did not present for the conclusion visit. Owners that presented their cat for the required follow-up visits and completed all online surveys received an incentive in the form of an account credit at the veterinary hospital.

### Outcomes of interest

BCS: Veterinarians practicing in the participating hospital network routinely assess body composition using a five-point BCS scale (1 = very thin, 2 = thin, 3 = ideal, 4 = overweight, 5 = obese). BCS was assessed at each visit and entered into the practice management system. For analysis, the five-point BCS scale was collapsed into three categories: ‘underweight’ (BCS < 3), ‘ideal’ (BCS = 3) or ‘overweight’ (BCS > 3).

Percentage change in body weight: All participating hospitals received a Health o meter 549KL Digital Pediatric Tray Scale (Sunbeam Products, Inc.) for weight measurement. Body weight was assessed at each visit and entered into the practice management system. For analysis, percentage change in body weight was calculated at each time point by subtracting weight at enrolment (*T*_0_) from the present weight (*T*_*x*_) and dividing by *T*_0_.

### Statistical analysis

Descriptive statistics (frequencies and proportions) of the weekly and monthly survey questions were calculated to summarise the survey data. The χ^2^ testing was performed to compare survey question response frequency between the treatment and control groups.

Because each cat completed multiple study visits, univariate and multivariate generalised linear models with repeated measurements were constructed using PROC MIXED (percentage weight change as outcome) and PROC GENMOD (BCS as outcome) procedures in Statistical Analysis Systems statistical software package version 9·3 (SAS Institute, Cary, NC, USA). Diet group (treatment or control), sex (male or female), age at enrolment and follow-up visits (2-week, 1–4 and 6 months) were included in the final models. A random effect for hospital was initially included in each model; however, because that variable had a negligible impact on the outcome, it was removed in the interest of parsimony. Possible two-way interactions were checked and included in the final model when they were statistically and clinically significant. *Post-hoc* comparisons (percentage weight change as outcome) were performed and OR (BCS as outcome) were calculated. Values of *P* ≤ 0·05 were considered statistically significant.

## Results

### Participants

A total of 253 cats were enrolled in the study, 125 cats into the treatment group and 128 into the control group. Of these, ninety cats in the treatment group (mean enrolment age of 5·2 (sd 0·7) months and mean enrolment weight of 2·8 (sd 0·7) kg) and ninety-seven cats in the control group (mean enrolment age of 5·2 (sd 0·8) months and mean enrolment weight of 2·9 (sd 0·6) kg) completed the study. Reasons for withdrawal from the study were similar between groups, with loss to follow-up as the most common (74 %), followed by unrelated pet illness (5 %) and unrelated pet death (5 %). Loss to follow-up was not significantly different between cats in the treatment group (twenty-three cats, 18 %) and cats in the control group (twenty-six cats, 20 %) (Pearson χ^2^ test *P* = 0·70).

### Outcomes

After controlling for enrolment age and sex, the odds of having a BCS of overweight assessed in the 6 months following neutering was greater for cats fed their standard diet than for cats fed the trial diet (OR 4·1; 95 % CI 2·1, 8·2; Table 1). Nine cats in the treatment group (10·0 %) and twenty cats in the control group (20·6 %) had a BCS of overweight at study completion. Mean completion weight was 4·4 (sd 0·9) kg for cats in the treatment group and 4·6 (sd 1·0) kg for cats in the control group. Percentage change in body weight differed significantly with enrolment age (*P* = 0·007; Table 2) and approached significance between diet groups (*P* = 0·08).

**Table 1.** Results of generalised linear mixed modelling of the probability of cats being assigned a body condition score (BCS) of overweight (BCS > 3) based on diet fed* in the 6 months following routine spay or neuter surgery (95 % confidence intervals (CI))

Variable	Comparison	OR	Lower CI	Upper CI	*P*
Diet	Control *v.* treatment	4·1	2·1	8·2	0·001
Sex	Neutered male *v.* spayed female	1·4	0·8	2·5	0·261
Enrolment Age	1 month difference	1·0	0·7	1·9	0·823

* Trial diet (*n* 90) *v.* standard diet (*n* 97).

**Table 2.** Difference between groups in mean percentage of weight gained by cats based on the diet fed* in the 6 months following routine spay or neuter surgery

Variable	Comparison	Difference	se	*P*
Diet	Control *v.* treatment	4·04	2·32	0·08
Sex	Neutered male *v.* spayed female	−1·41	2·35	0·55
Enrolment age	1 month difference	−5·17	1·52	0·007

* Trial diet (*n* 90) *v.* standard diet (*n* 97).

The average number of weekly online surveys completed for participants who finished the study was 23·2 (97 %); the average number of surveys completed was 23·7 (99 %) for the treatment group and 22·8 (95 %) for the control group. Based on χ^2^ analysis, owners of cats fed the trial diet were more likely than owners of cats fed their standard diet to report improvement in the previous month in coat gloss at months 1 (*P* = 0·003), 2 (*P* = 0·003), 3 (*P* = 0·004), 4 (*P* = 0·016) and 5 (*P* = 0·05) and more likely to report an increase in their cat begging for food at months 1 (*P* < 0·001), 2 (*P* < 0·001), 3 (*P* < 0·001) and 4 (*P* = 0·01). No difference between the two groups was identified for coat softness, coat aspect, shedding, dandruff, stool consistency or eagerness to eat.

## Discussion

The present study demonstrated in a real-world setting that a post-neutering weight control regimen coupled with a moderate fat, low-energy dry expanded diet can contribute to better weight control of client-owned cats in the months following neutering. In support of this conclusion was the significant (*P* ≤ 0·05) difference in the likelihood of cats being assessed as overweight when fed the treatment diet rather than their regular diet when controlling for other variables, and that finding was reinforced by the lower, albeit non-significant (*P* = 0·08), percentage weight gain in the treatment group. The sample size calculation for the present study did not take into account the need to control for multiple variables. Therefore, a larger sample size may have allowed identification of a significant difference in percentage weight gain between the groups if one truly existed.

Previous research in laboratory settings has established the importance of reducing cats' post-neutering energy intake through controlled feeding^(^^11^^)^ as well as the efficacy of feeding a low-energy-dense diet in decreasing post-neutering weight gain in cats^(^^7^^)^. However, methods that are successful under controlled laboratory conditions have the potential to be less effective in a real-world setting^(^^9^^)^. In particular, pet weight-loss and obesity prevention strategies are subject to a number of factors that influence compliance, including client perceptions of hunger^(^^12^^)^ and the use of non-standard measurement systems^(^^13^^)^.

Reduced incidence of overweight in the treatment group was observed despite a larger proportion of owners of cats fed the trial diet reporting their cat begging for food in the first 4 months after neutering than owners of cats fed their standard diet. Differences in perceived begging between the two groups may be indicative of differences in feeding compliance, which could be partially attributable to the fact that the trial diet came with strict feeding instructions whereas owners of cats fed their standard diet may have lacked similarly rigorous instructions. However, owner-perceived hunger has previously been linked to excessive feeding and owners' inability to cope with cats' hunger-related behaviours is a common cause of failure in feline weight loss programmes^(^^12^^)^. Negative impacts of begging behaviours in the treatment group, such as over feeding or lack of compliance, may have been mitigated by the fact that such begging behaviours were reported to subside after the fourth month. Owners feeding the trial diet may have also observed benefits beyond maintenance of body condition that encouraged them to remain compliant with the feeding protocol, as evidenced by a higher proportion of owners with cats fed the trial diet reporting improvement in their cat's coat gloss in the first 5 months after neutering.

Limitations of the present study include allocation of treatment at the hospital level, methods used for clinical assessment of body condition and subjective observation of compliance. Allocation of treatment to entire hospitals presents the possibility that unmeasured differences in provider, patient or pet-owner characteristics influenced the observed results. Randomisation by hospital was deemed superior to randomisation by patient because it prevented veterinarians from having patients in both the treatment and control groups, thereby avoiding a strong potential for contamination. The large number of hospitals and their random allocation to treatment groups is believed to have minimised the impact of inter-hospital differences. Assessment of body composition was limited to body weight and BCS. Although body weight provides a simple and relatively stable measure of body composition, it has little meaning when interpreted in isolation and can be impacted by dehydration or fluid accumulation^(^^14^^)^. BCS provides more comprehensive information about an animal's body composition, particularly when evaluated in conjunction with body weight^(^^15^^)^, but it is a subjective assessment that lacks the precision of quantitative approaches and is subject to issues of inter-observer variation^(^^14^^)^. Although more sophisticated techniques such as dual-energy x-ray absorptiometry would have provided more precise assessment of body composition, such procedures would have reduced the feasibility of the study. Another limitation was that feeding compliance and perceived impact of the trial diet were evaluated through online questionnaires completed by the owner using subjective impressions, and should be interpreted accordingly.

In summary, a post-neutering weight-control regimen coupled with a moderate fat, low-energy dry expanded diet significantly reduced the incidence of overweight in client-owned cats in the 6 months following neutering. Further research is warranted to determine whether the observed differences are maintained over time.
